# Long-Term Treatment Outcomes in Type 3 Neovascularization: Focus on the Difference in Outcomes between Geographic Atrophy and Fibrotic Scarring

**DOI:** 10.3390/jcm9041145

**Published:** 2020-04-16

**Authors:** Jae Hui Kim, Jong Woo Kim, Chul Gu Kim, Dong Won Lee

**Affiliations:** Department of Ophthalmology, Kim’s Eye Hospital, Konyang University College of Medicine, Seoul 150-034, Korea; kjwood@kimeye.com (J.W.K.); chulgukim@kimeye.com (C.G.K.); mediceye@kimeye.com (D.W.L.)

**Keywords:** type 3 neovascularization, retinal angiomatous proliferation, age-related macular degeneration, geographic atrophy, fibrotic scar, anti-vascular endothelial growth factor

## Abstract

Background: To evaluate the difference in the long-term treatment outcomes of type 3 neovascularization between eyes with geographic atrophy and those with fibrotic scars. Methods: This retrospective study included 195 eyes diagnosed with type 3 neovascularization and treated with anti-vascular endothelial growth factor (VEGF) agents. The included eyes were divided into three groups according to the fundus findings at the final visit: patients with fovea-involving geographic atrophy (GA group), patients with fovea-involving fibrotic scars (scar group), and patients with no fovea-involving geographic atrophy or fibrotic scars (non-GA/scar group). The best-corrected visual acuities (BCVA) of the three groups at the final visits were compared. Results: The mean follow-up period was 47.5 ± 20.7 months. The mean logMAR BCVA at the final visit was 1.18 ± 0.58 in the GA group (n = 58), 1.67 ± 0.58 in the scar group (n = 62), and 0.69 ± 0.64 in the non-GA/scar group (n = 75). The BCVA was significantly worse in the scar group than in the GA (*p* < 0.001) and the non-GA/scar groups (*p* < 0.001). Conclusion: Eyes with fibrotic scars showed the poorest visual outcomes in type 3 neovascularization among the studied groups. Preventing the development of fibrotic scars should be considered an important treatment goal.

## 1. Introduction

Neovascular age-related macular degeneration (AMD) is a sight-threatening disease, which may lead to progressive and profound visual deterioration [1,2]. Type 3 neovascularization is a subtype of neovascular AMD that is characterized by intraretinal neovascularization [3,4]. In 2001, Yannuzzi et al. reported cases of neovascular AMD characterized by intraretinal neovascularization and termed the condition as “retinal angiomatous proliferation” [3]. Later, Freund et al. expanded the spectrum of this peculiar form of neovascularization and introduced the term “type 3 neovascularization” [4]. 

Previously, neovascular AMDs were classified into two types, depending on the anatomical location of the lesion: subretinal pigment epithelial lesions were classified as type 1 neovascularization, and subretinal lesions were classified as type 2 neovascularization. Freund et al. suggested that distinct intraretinal lesions should be classified as type 3 neovascularization [4]. Intraretinal lesions were detected using angiography or optical coherence tomography (OCT) in early studies [3,4]. More recently, Li et al. provided actual histopathological evidence for the development of intraretinal neovascularization [5]. Currently, both of the terms “retinal angiomatous proliferation” and “type 3 neovascularization” are used to indicate neovascular AMD with intraretinal neovascularization.

The incidence of type 3 neovascularization was reported as 4.5%–15% of all neovascular AMD cases [6,7,8]. It usually develops in elderly women [7]. In addition, type 3 neovascularization is associated with a high incidence of drusens [9], reticular pseudodrusens [10], and very thin choroids [9]. Investigators have suggested that decreased perfusion to the outer retina may cause intraretinal neovascularization [9]. The incidence among different ethnicities is comparable [6,7].

Anti-vascular endothelial growth factor (VEGF) therapy is the mainstay of treatment for neovascular AMD [11]. Previously, type 3 neovascularization was considered refractory to treatment [12,13]. However, treatment outcomes have markedly improved after the advent of anti-VEGF therapy [14]. Nevertheless, frequent development of geographic atrophy (GA) in eyes with type 3 neovascularization is an important concern as it may lead to visual deterioration regardless of the response to treatment [15,16,17]. Moreover, the development of subretinal hemorrhages or retinal pigment epithelium (RPE) tears may cause abrupt vision loss during the treatment [18,19,20]. The long-term treatment outcomes of type 3 neovascularization have not yet been fully elucidated.

Established treatment regimens, such as the fixed dosing regimen, as-needed regimen with strict monthly follow-up, and treat-and-extend regimen, have shown excellent efficacies in the treatment of neovascular AMD [21,22,23]. In clinical practice, however, it is often not possible to apply a strict treatment protocol. Hence, treatment outcomes in real-world settings [24,25] are generally unfavorable compared to those observed in clinical trials. Nevertheless, real-world evidence has its value and may contribute to the understanding of the benefits and the risks of therapies [26].

The purpose of the present study was to evaluate the long-term treatment outcomes of anti-VEGF therapy for type 3 neovascularization in a real-world setting. We focused on the difference between the visual outcomes in eyes with GA and those with fibrotic scars.

## 2. Materials and Methods

The study was approved by the Institutional Review Board (Kim’s Eye Hospital IRB, No. 2019-11-005).

### 2.1. Patients

The inclusion criteria for the study were as follows: (1) diagnosis of type 3 neovascularization between January 2010 and July 2017; (2) treatment with 3 loading injections of anti-VEGF after initial diagnosis; and (3) follow-up duration of 24 months or longer. The exclusion criteria were as follows: (1) history of treatment for type 3 neovascularization; (2) other retinal vascular disorders; (3) the presence of definite chorioretinal anastomosis on fundus photography; and (4) the presence of a fovea-involving fibrotic scar. If both eyes satisfied the eligibility criteria, the eye with earlier symptoms was included. A part of this patient cohort was also included in our previous studies [19,27,28].

### 2.2. Examinations

At diagnosis, the best-corrected visual acuity (BCVA) was measured and fundus photographs were obtained. Fluorescein angiography and OCT were performed in all the patients. Indocyanine-green angiography (ICGA) was performed in selected cases, at the discretion of the physician. Type 3 neovascularization was diagnosed using multimodal imaging based on the previously suggested method [29] The staging of type 3 neovascularization lesions was performed based on OCT findings [30]. Fibrotic scars [31] and GA were identified using fundus photographs and OCT images. Central foveal thickness was defined as the retinal thickness at the fovea.

### 2.3. Treatment and Follow-Up

The treatment and follow-up methods used in this study were similar to those in our previous study [32]. Patients were initially administered 3 monthly injections. Ranibizumab (0.5 mg/0.05 mL of Lucentis^TM^; Genentech Inc., San Francisco, CA, USA) or aflibercept (2.0 mg/0.05 mL of Eylea^TM^; Regeneron, Tarrytown, NY, USA) was used for the initial treatments. After an initial treatment, re-treatment was performed on an as-needed basis. One of 3 anti-VEGF agents—ranibizumab, aflibercept, or bevacizumab (1.25 mg/0.05 mL of Avastin^TM^; Genentech Inc., San Francisco, CA, USA)—was used for the additional treatment. If the treating physician determined that a more effective treatment was required to preserve vision, the treatment regimen was changed from the as-needed regimen to the proactive regimen. In some patients, treatment was discontinued at the physician’s discretion.

### 2.4. Outcome Measures

The BCVA values measured at diagnosis were compared with those at 3 months (1 month after the 3 loading injections), 12 months, 24 months, and the final visit. If a patient did not visit the hospital at exactly 12 or 24 months, values measured at the visits closest to 12 or 24 months were used for the analysis. Additionally, time-dependent change in the proportion of eyes experiencing irreversible visual deterioration to 20/200 or worse was evaluated. In this analysis, the proportion was first estimated after 3 loading injections.

**Figure 1:**
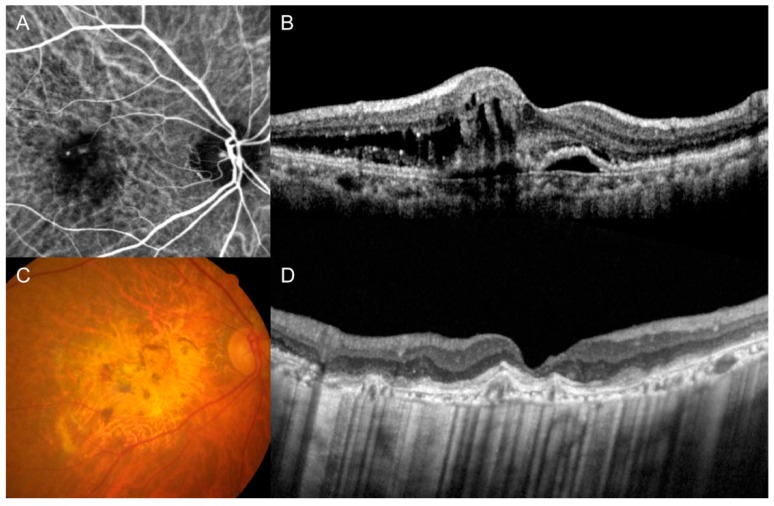
Indocyanine-green angiography (**A**), optical coherence tomography (**B**,**D**), and fundus photography (**C**) images of representative cases showing fovea-involving geographic atrophy. Images were taken at diagnosis (**A**,**B**) and final follow-up (**C**,**D**).

**Figure 2:**
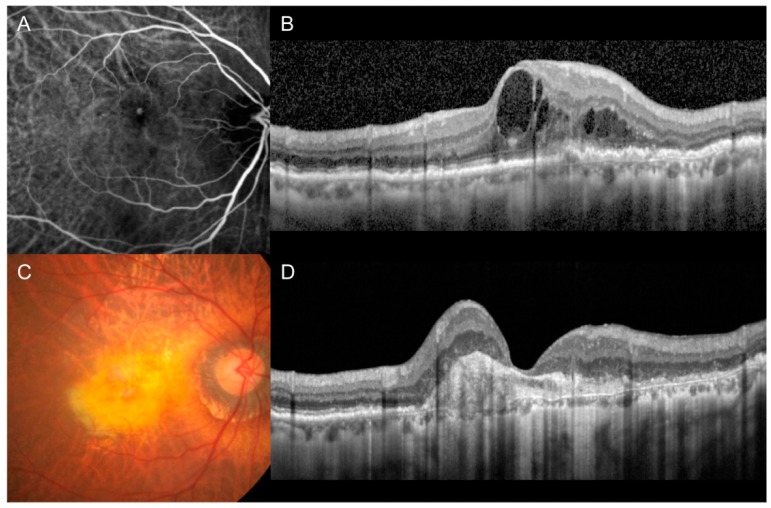
Indocyanine-green angiography (**A**), optical coherence tomography (**B**,**D**), and fundus photography (**C**) images of representative cases showing fovea-involving fibrotic scars. Images were taken at diagnosis (**A**,**B**) and final follow-up (**C**,**D**).

Patients were divided into 3 groups according to the fundus photography and OCT findings at the final visit: patients exhibiting fovea-involving GA (GA group; Figure 1), patients exhibiting fovea-involving fibrotic scars (scar group; Figure 2), and patients exhibiting no fovea-involving GA or fibrotic scars (non-GA/scar group). In cases where the fibrotic scar was eventually replaced by GA [33], the eye was included in the GA group. When accurate fundus visualization was not possible due to vitreous hemorrhage (VH) at the final visit, the classification was performed based on the fundus findings before the development of VH. Follow-up period, the BCVA at diagnosis and at the final visit, and the amount of change in the BCVA from the diagnosis to the final visit of the 3 groups were compared.

Additional analyses were performed to identify differences in patient characteristics between the GA group, the scar group, and the non-GA/scar group. Age, sex, diabetes mellitus, hypertension, lens status, stage of the disease, the incidence of reticular pseudodrusens, type of anti-VEGF agent used for the loading injections, and the number of anti-VEGF injections of the 3 groups were compared. The association between these baseline characteristics and changes in BCVA throughout the follow-up period was also analyzed.

In the eyes that developed subretinal hemorrhage of 1 disc area or greater during the follow-up, the incidences of GA and fibrotic scars were estimated. The proportions of GA, scars, and non-GA/scars in eyes treated with either ranibizumab monotherapy (ranibizumab group) or aflibercept monotherapy (aflibercept group) were compared. In the non-GA/scar group, the associations between these baseline characteristics and changes in BCVA throughout the follow-up period were also analyzed.

The BCVA values were measured using the decimal visual acuity chart and then converted to the logarithm of minimal angle of resolution (logMAR) values for the analysis. According to the recommendation by Holladay [34], counting fingers and hand motion visual acuities were converted to logMAR values 2 and 3, respectively. There is no established method to convert the light-perception visual acuity to logMAR for statistical analysis. In this study, however, light-perception was also converted to a logMAR value of 3 (equivalent to the hand motion visual acuity) for the statistical analysis.

### 2.5. Statistical Analyses

Statistical analyses were performed using SPSS (Version 12.0 for Windows; IBM Corporation, Armonk, NY, USA). BCVAs were compared at different time points using repeated-measures analysis of variance, and individual comparisons were performed using Bonferroni’s method. Comparisons among the 3 groups were performed using a one-way analysis of variance (continuous variables) with or without Tukey’s test or chi-squared test (nominal variables). The association between baseline characteristics and changes in BCVA was analyzed using multivariate linear regression. *p*-values less than 0.05 were considered statistically significant.

## 3. Results

During the study period, 195 eyes from 195 patients (42 men and 153 women) met the inclusion criteria. The mean age of the patients was 75.7 ± 6.0 years (mean ± standard deviation). Table 1 summarizes the baseline characteristics of the included patients. At diagnosis, 26 patients (13.3%) had bilateral type 3 neovascularization. Of them, the included eye was the better-seeing eye in nine. One hundred forty-nine eyes (76.4%) were initially treated with ranibizumab, and the remaining 46 eyes (23.6%) were initially treated with aflibercept. The anti-VEGF agents used during the entire follow-up period were ranibizumab alone (77 eyes), aflibercept alone (37 eyes), ranibizumab and bevacizumab (59 eyes), aflibercept and bevacizumab (9 eyes), ranibizumab and aflibercept (5 eyes), and ranibizumab, aflibercept, and bevacizumab (8 eyes).

The mean follow-up period was 47.5 ± 20.7 months. During the follow-up period, 9.5 ± 4.8 anti-VEGF injections were administered. The mean number of injections administered during the first year, the second year, and between the second year and the final visit was 4.5 ± 1.3, 2.2 ± 1.8, and 1.8 ± 3.0, respectively. Sixteen eyes underwent cataract surgery, and five eyes underwent vitrectomy. Both cataract surgery and vitrectomy were performed in two eyes. At the final follow-up, fundus findings could not be assessed accurately in seven eyes due to dense VH. Since vitrectomy was not performed on these eyes, the identification of GA and scar was based on the fundus findings before the development of VH. Of them, three, two, and two eyes had counting fingers, hand motion, and light-perception visual acuities, respectively. Out of the 195 eyes, treatment was discontinued in 47 (24.1%).

**Figure 3:**
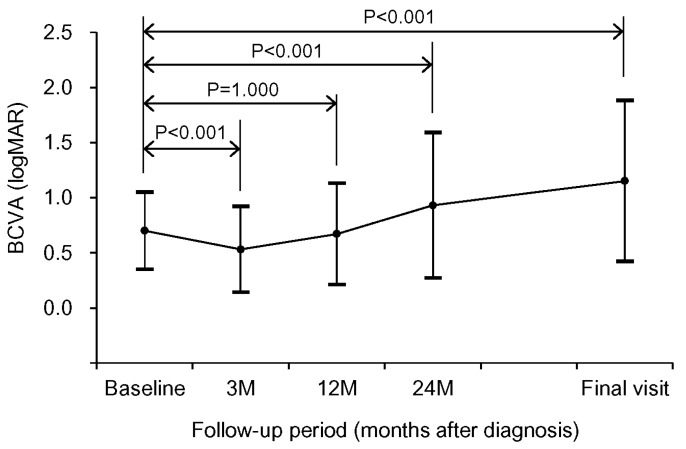
Time-dependent changes in best-corrected visual acuity (BCVA). Statistical analysis was performed using repeated-measures analysis of variance. Individual comparisons were performed using Bonferroni’s method. logMAR: logarithm of minimal resolution, M: month.

**Figure 4:**
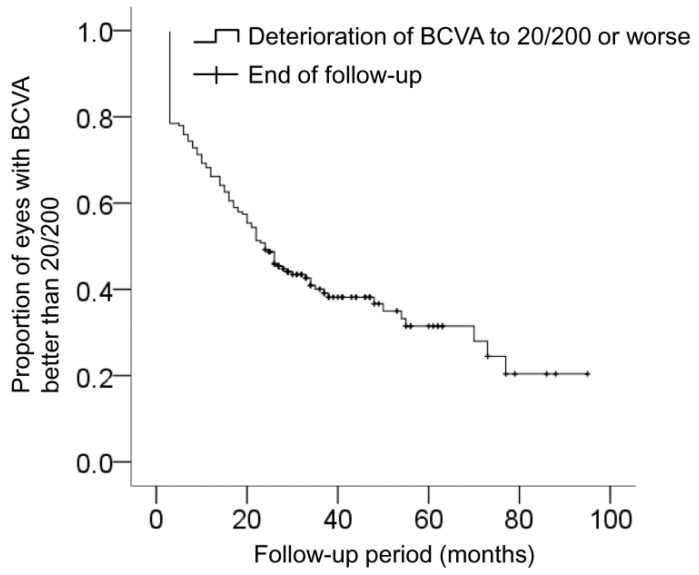
Kaplan–Meier graph showing changes in the proportion of eyes with best-corrected visual acuity (BCVA) better than 20/200 according to the follow-up period.

The mean logMAR BCVA was 0.70 ± 0.35 (Snellen equivalent = 20/100) at diagnosis, 0.53 ± 0.39 (20/67) at 3 months, 0.67 ± 0.46 (20/93) at 12 months, 0.93 ± 0.66 (20/176) at 24 months, and 1.15 ± 0.73 (20/282) at the final visit (Figure 3). When compared with the baseline value, the BCVA significantly improved at 3 months (*p* < 0.001), but was not significantly different at 12 months (*p* = 1.000). The BCVA values at 24 months (*p* < 0.001) and at the final follow-up (*p* < 0.001) showed significant deterioration compared to the baseline values. Compared to the baseline value, a 3-line or greater (≥0.3 logMAR value) improvement in the BCVA was noted in 27 eyes (13.8%) at the final visit. A 3-line or greater deterioration in the BCVA was noted in 112 eyes (57.4%). The BCVA remained stable in the remaining 56 eyes (28.7%). The logMAR BCVA was 1.00 (20/200) or worse in 71 eyes (36.4%) at diagnosis and in 120 eyes (61.5%) at the final visit. Figure 4 shows the time-dependent changes in the proportion of eyes with logMAR BCVA better than 1.00 (20/200). The mean estimated interval between the diagnosis and the deterioration of logMAR BCVA to 1.00 (20/200) or worse was 39.3 ± 2.9 months. 

After the division into 3 groups according to the presence of GA or fibrotic scar at the final visit, 58 eyes (29.7%) were included in the GA group, 62 eyes (31.8%) were included in the scar group, and the remaining 75 eyes (38.5%) were included in the non-GA/scar group. The mean follow-up duration was 51.6 ± 20.1 months in the GA group, 52.9 ± 23.4 months in the scar group, and 39.9 ± 16.4 months in the non-GA/scar group. The follow-up duration was significantly shorter in the non-GA/scar group than in the GA (*p* = 0.003) and the scar groups (*p* = 0.001). There was no significant difference between the follow-up durations of the GA and the scar groups (*p* = 0.924).

Comparisons of BCVA among these three groups are shown in Table 2. At diagnosis, no significant difference was observed between the BCVAs of the GA and the scar groups (*p* = 0.395). At the final visit, the BCVA was significantly better in the GA group than in the scar group (*p* < 0.001). A significantly greater degree of visual deterioration was noted in the scar group compared to the GA group (*p* < 0.001). At diagnosis, the proportion of eyes exhibiting a BCVA of 20/200 or worse was 37.9% in the GA group, 51.6% in the scar group, and 22.7% in the non-GA/scar group. At the final visit, the proportion was 68.9% in the GA group, 98.4% in the scar group, and 28.0% in the non-GA/scar group.

**Figure 5:**
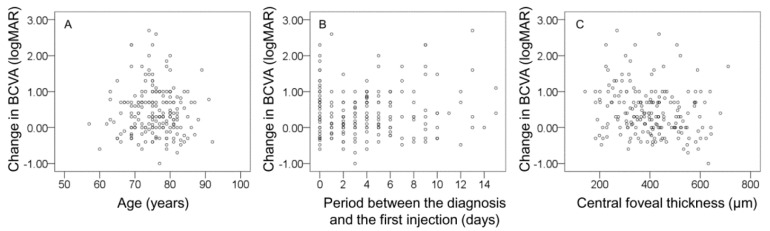
Scatterplots showing the distribution of changes in BCVA with age during the follow-up period (**A**), the period between the diagnosis and the first injection (**B**), and baseline central foveal thickness (**C**). Positive values indicate deterioration in BCVA, and negative values indicate improvement in BCVA.

Figure 5 shows the distribution of changes in BCVA with age, the period between the diagnosis and the first injection, and the baseline central foveal thickness.

The results of the comparisons between the GA group, the scar group, and the non-GA/scar group are summarized in Table 3. There were significant differences in the stage of disease (*p* = 0.024) and number of anti-VEGF injections (*p* = 0.013) of the three groups. Other characteristics, including age (*p* = 0.787), sex (*p* = 0.228), diabetes mellitus (*p* = 0.361), hypertension (*p* = 0.538), lens status (*p* = 0.729), reticular pseudodrusen (*p* = 0.331), and type of anti-VEGF agent used for the loading injections (*p* = 0.093), were not significantly different. During the follow-up period, treatment was discontinued in five eyes (8.6%) in the GA group and 38 eyes (61.3%) in the scar group.

Thirty-one eyes (15.9%) developed subretinal hemorrhage of one disc area or greater during the follow-up. Among these, fovea-involving fibrotic scar eventually developed in 25 eyes (80.6%) and fovea-involving GA developed in 2 eyes (6.5%). In the ranibizumab group (n = 77), 27 eyes (35.1%) had GA and 25 eyes (32.5%) had fibrotic scar. GA or fibrotic scar were not noted in the remaining 25 eyes (32.5%). In the aflibercept group (n = 37), 15 eyes (40.5%) had GA and 5 eyes had fibrotic scars (13.5%). GA or fibrotic scars were not noted in the remaining 17 eyes (45.9%). There was no significant difference in the incidence between the two groups (*p* = 0.089).

In the multivariate analysis, no baseline characteristic was significantly associated with changes in visual acuity throughout the follow-up period (Supplementary Materials Table S1). In the non-GA/scar group, no baseline characteristic was significantly associated with changes in visual acuity throughout the follow-up period (Supplementary Materials Table S2).

Of the 118 phakic eyes at diagnosis, cataract surgery was performed for 18 during the follow-up period. Of the 43 cases of stage 2 disease at diagnosis, 30 progressed to stage 3 disease. Of the 51 eyes without pseudodrusens at diagnosis, none showed pseudodrusen formation.

## 4. Discussion

Several studies have reported the long-term treatment outcomes of anti-VEGF therapy for type 3 neovascularization [14,15,16,17,35,36,37,38,39,40]. However, in most of those studies, a relatively small number of patients were included [14,16,17,36,37,38,39,40], and the follow-up periods were no more than three years [14,15,16,36,37,38,39,40]. Hence, the long-term treatment outcomes of type 3 neovascularization merit further investigation.

Previous studies that investigated the real-world treatment outcomes of neovascular AMD have shown a typical pattern of visual acuity change. The visual acuity improved during the first several months after treatment initiation but continuously deteriorated thereafter [24,25]. Visual acuity changes in our patients showed a similar trend. There was a significant improvement in the BCVA after three initial loading injections. However, the BCVA at 12 months was not significantly different from the baseline value. The values at 24 months and the final visits were significantly worse than the baseline values. As a result, 61.5% of the eyes eventually had visual acuities of 20/200 or worse.

Despite these unfavorable long-term visual outcomes, it is noteworthy that the visual outcomes in eyes without GA or fibrotic scars were markedly better than those in eyes with these findings. Visual acuity in the non-GA/scar group was relatively stable throughout the follow-up period. We observed a slight decrease in the mean logMAR BCVA values (0.58 ± 0.34 at diagnosis to 0.69 ± 0.64 at the final visit) despite the relatively long follow-up period (mean duration = 39.9 months). This suggests that in the absence of GA or a fibrotic scar, vision in the eyes with type 3 neovascularization can be preserved for a considerably long period.

GA and scar are well-known primary causes of poor treatment outcomes in neovascular AMD [41,42]. In our patients, these findings eventually developed in approximately two-thirds of the included eyes. GA is characterized by loss of choriocapillaris, RPE, and retinal outer layers [43,44]. GA is one of the important points of consideration while treating type 3 neovascularization, as eyes with type 3 neovascularization are at a high risk of GA development [45]. The reported incidence of GA ranges between 23.8% and 60% after anti-VEGF therapy for type 3 neovascularization [40,46,47,48,49]. In this study, fovea-involving GA developed in 29.7% of the included eyes. Significant deterioration of visual acuity was noted in eyes with GA, and only 31.1% of the eyes had visual acuities better than 20/200 at the final visit.

Subretinal fibrosis, which is responsible for the formation of fibrotic scar, is a result of a wound healing response that follows choroidal neovascularization [50]. Diffuse loss of photoreceptors is often noted in scars of 200 µm or more in thickness [51]. In the Comparison of Age-Related Macular Degeneration Treatment Trials, the cumulative proportions of eyes with fibrotic scars were 32%, 46%, and 56% at year 1, 2, and 5, respectively [33]. At 2 years, the adjusted mean visual acuity score was worse in eyes with fibrotic scars than in those with other pathologies in the foveal center [42]. In this study, fovea-involving fibrotic scars were noted in 31.8% of the eyes. The visual outcomes in eyes with fibrotic scars were extremely poor, with visual acuities of 20/200 or worse at the final visit in almost all these eyes.

Poor visual prognosis in our patients with GA or fibrotic scars is somewhat expected. However, one notable finding was that there was a marked difference between the visual outcomes of the GA and the scar groups. More specifically, the visual outcomes were markedly worse in the scar group than in the GA group. Because of the high incidence of GA in type 3 neovascularization, GA is considered an important issue in the treatment of type 3 neovascularization [15,17,23]. Although some GA lesions are related to low incidence of re-activation [27], GA is generally believed to be associated with poor visual outcomes [15,17]. Freund et al. suggested the need for studies to determine whether type 3 neovascularization can be managed more safely with the as-needed regimen, rather than the treat-and-extend regimen, by reducing the risk of GA [23]. Nevertheless, the more devastating visual prognosis in the scar group found in this study suggests that preventing the development of fibrotic scar would be as important as, or even more important than, preventing the development of GA. To the best of our knowledge, no previous study has focused on the method to prevent the development of fibrotic scars in type 3 neovascularization.

To address this issue, we focused on eyes with subretinal hemorrhage that had very high risks of fibrotic scar development. The development of a subretinal hemorrhage is a catastrophic event in type 3 neovascularization. It may cause abrupt vision loss [19]. In addition, profound vision loss frequently occurs despite treatment in eyes with subretinal hemorrhage [18,20]. Despite this characteristic clinical course, macular morphology after the hemorrhage has not yet been fully elucidated. In this study, fibrotic scars eventually developed in 80.6% of the eyes with subretinal hemorrhage and approximately 40% of the fibrotic scars (25 of 62 cases) developed in eyes with subretinal hemorrhage. This suggests that preventing the development of hemorrhage may contribute to the reduction in the incidence of fibrotic scars in type 3 neovascularization. One interesting finding from previous studies is that the hemorrhage rarely develops when using the treat-and-extend regimen [35,52]. Based on this result, we postulate that using the treat-and-extend regimen might show some benefit in preventing fibrotic scarring by reducing the risk of subretinal hemorrhage. Bloch et al. demonstrated that subretinal fibrosis is associated with a longer interval between diagnosis and treatment [53]. This may suggest that the prolonged damage to the retina caused by uncontrolled neovascularization is an important contributor to scar development. Since the treat-and-extend regimen is characterized by long-term continuous suppression of VEGF, it might minimize the retinal damage due to the re-activation of the lesion. If our postulation is valid, it might highlight the benefit of the treat-and-extend regimen in type 3 neovascularization despite the concerns regarding GA. However, proving this postulation is beyond the scope of the present study. Further studies comparing the incidence of fibrotic scar between the treat-and-extend regimen and the other treatment regimens are required to verify this theory.

In this study, significant differences in the stage of disease and the number of anti-VEGF injections were observed among the GA group, the scar group, and the non-GA/scar group. The incidence of stage 2 disease was higher in the GA group than in the other 2 groups. In a previous classification based on ICGA findings by Yannuzzi et al., stage 3 disease was defined as the presence of chorioretinal anastomosis [3]. More recently, Su et al. suggested an OCT-based classification [30]. In this classification, the presence of pigment epithelial detachment (PED) on OCT was considered as a hallmark of stage 3 disease. In this study, cases were classified using the method suggested by Su et al. [30]. In a previous study with a 1-year follow-up, stage 3 disease showed worse visual outcomes than stage 2 disease [54]. In addition, submacular hemorrhage developed only in stage 3 diseases [54]. It is likely that the higher proportion of stage 3 diseases in the fibrotic scar group may be associated with the more aggressive nature of stage 3 diseases. The exact reason the proportion of stage 3 disease is also relatively higher in the non-GA/scar group is not clear. We postulate that the significantly shorter follow-up duration in the non-GA/scar group compared to the other 2 groups may have affected this result. That is, GA or fibrotic scars may eventually develop in some of the eyes in the non-GA/scar group when patients are followed-up for longer. For this reason, it is not certain whether stage 3 disease is associated with the absence of GA or fibrotic scars. The present study is retrospective, and the confounding factors were not controlled. More controlled studies are needed to elucidate the influence of the stage of disease on the development of GA or fibrotic scars.

The number of anti-VEGF injections was relatively lower in the GA group than in the other 2 groups. Previous studies have shown that the development and progression of GA are associated with decreased lesion activity [27,47]. Our result is in line with those of the previous studies. 

Aflibercept was more frequently used for the initial loading injections in the GA group than in the scar group. There is no clear evidence suggesting a difference in the risk of GA or fibrotic scars among different anti-VEGF agents. Gillies et al. attempted to reveal a difference in the risk of GA between ranibizumab and aflibercept [55]. However, the GA outcome of the study has not yet been reported. In this study, anti-VEGF agents were selected according to the physician’s preference. Moreover, three different anti-VEGF agents were used. Hence, the effect of a single agent on the treatment outcome could not be accurately evaluated. Further studies with long-term treatment using a single anti-VEGF agent are needed to accurately identify the effect of different agents on the development of GA and fibrotic scar.

The exact reason the scar group showed worse visual outcomes than the GA group is unknown. In the study by Sharma et al. [42], 2-year visual acuities of eyes with scars and those with GA, hemorrhage, RPE tear, or blocked fluorescence were comparable. However, the visual outcomes in eyes with GA only were not separately analyzed. To date, no study has directly compared the outcomes between eyes with scars and those with GA. We postulate the causes of the difference in visual outcomes between the scar group and the GA group as follows. 

The development of a scar has been considered to be associated with extensively damaged and scattered RPE [50]. Scar development is associated with subretinal lesions such as subretinal hemorrhage [56], subretinal hyperreflective material [31], and classic choroidal neovascularization [31]. The common pathology of these findings is that they directly damage the outer retinal layers, including the photoreceptor layer and RPE layer. It is possible that the retinal outer layers were severely damaged before the development of the scar. The development of a scar may further damage the outer layer tissues by blocking oxygen and nutrition supply from the choroid. In general, the degeneration of retinal tissue due to GA gradually progresses from mild to severe stages [57]. For this reason, the eyes in the GA group may have had different residual visual functions, and in some patients, visual function may have been relatively preserved despite GA development.

In this study, the number of anti-VEGF injections was relatively low despite the long follow-up duration. Moreover, the injection frequency markedly decreased over time. The primary reason for this low injection frequency may be that strict monthly follow-up was not performed when using the as-needed regimen, resulting in undertreatment in some patients. In addition, treatment was eventually discontinued in 24.1% of the included patients. Moreover, GA development may partially contribute to the lower injection frequency, especially in the later follow-up period.

In this study, 60.5% of the patients were phakic at diagnosis. Previous studies have shown that the lens status may not influence the treatment outcomes of neovascular AMD [58,59]. In our patients, there was no significant difference in the phakic status among eye groups with different fundus findings. In the multivariate analysis, no baseline characteristic was significantly associated with changes in visual acuity. Since our patients were followed up for a relatively long period, numerous uncontrolled factors may have affected visual acuity. For this reason, a variable-controlled study is needed to validate our results.

The present study has certain limitations. This study was retrospective, and analyses were based on the data from a real-world setting. The treatment methods were not strictly controlled, and the treatment regimens varied at the discretion of the treating physician. Hence, some of our patients may have been undertreated. Only a mean of 9.5 anti-VEGF injections was administered despite the mean 47.5-month follow-up period. The influence of undertreatment on the study results may not be negligible. Light-perception visual acuity is not measurable [34]. In this study, however, it was arbitrarily converted to logMAR value 3, which is equivalent to hand motion visual acuity. We believe that this may not have significantly influenced the study results, as light-perception visual acuity was noted in only two eyes. However, using an arbitrary method is an obvious limitation of the study. Visual acuity at the final visit could not be accurately assessed in seven eyes due to VH. The resolution of VH may improve vision in some eyes. In this study, the incidence of diabetes mellitus (25.1%) was relatively higher than that in the healthy elderly Korean population (18%–24%) [60]. Although several investigators reported the association between diabetes mellitus and late AMD [61], it is not certain whether the presence of diabetes mellitus influences the treatment outcomes of neovascular AMD. Nevertheless, this possibility may not be completely denied. In this study, the proportion of female patients (78.5%) was higher than that of male patients (21.5%). It is reported that type 3 neovascularization usually develops in elderly women. The proportion of women has been reported to be 69.1%–77.0% in previous studies [15,20,30]. Thus, we believe that the difference in the sex ratio may not have significantly contributed to bias in the study results. At diagnosis, the BCVA was 20/200 or worse in 36.4% of the included eyes, suggesting that these eyes may have had a relatively long-standing disease. Thus, the study result may not reflect the outcomes following early detection and treatment. In this study, pseudodrusen development was not noted during the follow-up period. However, it is possible that the development of GA, scar, or hemorrhage may have precluded the accurate identification of pseudodrusen and may have led to under-identification of new pseudodrusen development. Lastly, sampling/experimental design was not performed before the study was conducted, and sample size was also not calculated. Thus, the exact power of the statistical analyses could not be verified.

In conclusion, we evaluated the long-term treatment outcomes of type 3 neovascularization in a real-world setting. Visual acuity improved after the initial treatment, but continuously deteriorated afterwards. As a result, significant visual deterioration was noted when compared with the baseline values after the second year. The development of fovea-involving GA and fibrotic scars were associated with poor visual outcomes. However, eyes with fibrotic scars showed markedly worse visual outcome than in those with GA. This result suggests that preventing the development of fibrotic scars should be an important treatment goal in type 3 neovascularization.

## Figures and Tables

**Table 1:** Baseline characteristics of the included patients (n = 195).

Characteristics	Values
Age, years	75.7 ± 6.0
Sex, men:women	42 (21.5%):153 (78.5%)
Diabetes mellitus	49 (25.1%)
Hypertension	111 (56.9%)
Phakia at diagnosis	118 (60.5%)
Stage of the disease	
Stage 2	43 (22.1%)
Stage 3	152 (77.9%)
Pseudodrusen	144 (73.8%)
Type of anti-VEGF agent used for the loading injections	
Ranibizumab	149 (76.4%)
Aflibercept	46 (23.6%)
Best-corrected visual acuity, logMAR (Snellen equivalent)	0.70 ± 0.35 (20/100)

The data are presented as the mean ± standard deviation or number (percentage). logMAR: logarithm of minimal angle of resolution, VEGF: vascular endothelial growth factor.

**Table 2:** Comparison of best-corrected visual acuities of the geographic atrophy (GA), scar, and non-GA/scar groups.

BCVA (logMAR)	GA Group (n = 58)	Scar Group (n = 62)	Non-GA/Scar Group (n = 75)	*p*-Value
At diagnosis	0.73 ± 0.35	0.81 ± 0.32	0.58 ± 0.34	<0.001 *
	a ^†^	a ^†^	b ^†^	
At the final visit	1.18 ± 0.58	1.67 ± 0.58	0.69 ± 0.64	<0.001 *
	a ^†^	b ^†^	c ^†^	
Change in BCVA	0.44 ± 0.56	0.86 ± 0.62	0.11 ± 0.57	<0.001 *
	a ^†^	b ^†^	c ^†^	

Data are presented as the mean ± standard deviation. BCVA: best-corrected visual acuity, logMAR: logarithm of minimum angle of resolution. * Statistical analysis was performed using one-way analysis of variance. ^†^ Statistical analysis was performed using the one-way analysis of variance with a Tukey test. The same letter indicates a non-significant difference between groups, whereas different letters indicate significant differences between groups.

**Table 3:** Comparison of characteristics among the geographic atrophy (GA), the scar, and the non-GA/scar groups.

Characteristics	GA Group (n = 58)	Scar Group (n = 62)	Non-GA/Scar Group (n = 75)	*p*-Value
Age, years	75.3 ± 5.6	75.5 ± 5.2	75.9 ± 6.8	0.787 *
Sex				0.228 ^†^
Men	8 (13.7%)	15 (24.2%)	19 (25.3%)	
Women	50 (86.2%)	47 (75.8%)	56 (74.7%)	
Diabetes mellitus	13 (22.4%)	13 (20.9%)	23 (30.7%)	0.361 ^†^
Hypertension	30 (51.7%)	35 (56.5%)	46 (61.3%)	0.538 ^†^
Phakia at diagnosis	36 (62.1%)	35 (56.5%)	47 (62.7%)	0.729 ^†^
Stage of disease				0.024 ^†^
Stage 2	20 (34.5%)	10 (16.1%)	13 (17.3%)	
Stage 3	38 (65.5%)	52 (83.9%)	62 (82.7%)	
Reticular pseudodrusen	47 (81.0%)	44 (70.9%)	53 (70.7%)	0.331 ^†^
Type of anti-VEGF agent used for the loading injections				0.093 ^†^
Ranibizumab	40 (68.9%)	53 (85.5%)	56 (74.7%)	
Aflibercept	18 (31.0%)	9 (14.5%)	19 (25.3%)	
No. of anti-VEGF injections	7.2 ± 4.9	8.6 ± 4.2	9.5 ± 4.9	0.013 *

Data are presented as the mean ± standard deviation or number (percentage). VEGF: vascular endothelial growth factor. * Statistical analysis was performed using one-way analysis of variance. ^†^ Statistical analysis was performed using the chi-squared test.

## Supplementary Materials

The following are available online at https://www.mdpi.com/2077-0383/9/4/1145/s1, Table S1: Association of baseline characteristics with changes in best-corrected visual acuity throughout the follow-up period; Table S2: Association between baseline characteristics and changes in best-corrected visual acuity throughout the follow-up period in the non-GA/scar group.
