# Supplementary material for: Long-Term Treatment Outcomes in Type 3 Neovascularization: Focus on the Difference in Outcomes between Geographic Atrophy and Fibrotic Scarring
Source: J Clin Med. 2020 Apr 16;9(4):1145. doi: 10.3390/jcm9041145 (PMC7230588; doi:10.3390/jcm9041145)
Supplement: Supplementary file 1 [file jcm-09-01145-s001.pdf]

Table S1. Association of baseline characteristics with changes in best-corrected visual acuity throughout the follow-up period.

Characteristics	P-value	*β*
Age	0.688	−0.030
Sex	0.844	−0.015
Diabetes mellitus	0.195	0.097
Hypertension	0.232	−0.091
Phakia at diagnosis	0.301	0.078
Stage of disease	0.235	−0.087
Reticular pseudodrusen	0.159	−0.111
Type of anti-VEGF agent used for the loading injections	0.098	−0.121
Best-corrected visual acuity	0.812	−0.018

* Statistical analysis was performed using multivariate linear regression.

Table S2. Association between baseline characteristics and changes in best-corrected visual acuity throughout the follow-up period in the non-GA/scar group.

Characteristics	P-value	*β*
Age	0.590	0.070
Sex	0.825	−0.031
Diabetes mellitus	0.093	0.233
Hypertension	0.792	−0.035
Phakia at diagnosis	0.371	0.121
Stage of disease	0.574	0.069
Reticular pseudodrusen	0.675	−0.059
Type of anti-VEGF agent used for the loading injections	0.372	−0.111
Best-corrected visual acuity	0.308	0.130

* Statistical analysis was performed using multivariate linear regression.
